# Changes in Dietary Fat Intake and Projections for Coronary Heart Disease Mortality in Sweden: A Simulation Study

**DOI:** 10.1371/journal.pone.0160474

**Published:** 2016-08-04

**Authors:** Lena Björck, Annika Rosengren, Anna Winkvist, Simon Capewell, Martin Adiels, Piotr Bandosz, Julia Critchley, Kurt Boman, Maria Guzman-Castillo, Martin O’Flaherty, Ingegerd Johansson

**Affiliations:** 1 Department of Molecular and Clinical Medicine, Sahlgrenska Academy, Gothenburg University, Gothenburg, Sweden; 2 Institute of Health and Care Sciences, Sahlgrenska Academy, Gothenburg University, Gothenburg, Sweden; 3 Department of Internal Medicine and Clinical Nutrition, Sahlgrenska Academy, University of Gothenburg, Gothenburg, Sweden; 4 Division of Public Health, University of Liverpool, Liverpool, United Kingdom; 5 Centre for Applied Biostatistics, Occupational and Environmental Medicine, University of Gothenburg, Gothenburg, Sweden; 6 St George's, University of London, Population Health Research Institute, Cranmer Terrace, London, United Kingdom; 7 Research Unit, Medicine-Geriatric, Skellefteå, Department of Public Health and Clinical Medicine, Umeå University, Umeå, Sweden; 8 Department of Odontology, Umeå University, Umeå, Sweden; Garvan Institute of Medical Research, AUSTRALIA

## Abstract

**Objective:**

In Sweden, previous favourable trends in blood cholesterol levels have recently levelled off or even increased in some age groups since 2003, potentially reflecting changing fashions and attitudes towards dietary saturated fatty acids (SFA). We aimed to examine the potential effect of different SFA intake on future coronary heart disease (CHD) mortality in 2025.

**Methods:**

We compared the effect on future CHD mortality of two different scenarios for fat intake a) *daily SFA intake decreasing to 10 energy* percent *(E%)*, and b) *daily SFA intake rising to 20 E%*. We assumed that there would be moderate improvements in smoking (5%), salt intake (1g/day) and physical inactivity (5% decrease) to continue recent, positive trends.

**Results:**

In the baseline scenario which assumed that recent mortality declines continue, approximately 5,975 CHD deaths might occur in year 2025. Anticipated improvements in smoking, dietary salt intake and physical activity, would result in some 380 (-6.4%) fewer deaths (235 in men and 145 in women). In combination with a mean SFA daily intake of 10 E%, a total of 810 (-14%) fewer deaths would occur in 2025 (535 in men and 275 in women). If the overall consumption of SFA rose to 20 E%, the expected mortality decline would be wiped out and approximately 20 (0.3%) additional deaths might occur.

**Conclusion:**

CHD mortality may increase as a result of unfavourable trends in diets rich in saturated fats resulting in increases in blood cholesterol levels. These could cancel out the favourable trends in salt intake, smoking and physical activity.

## Introduction

Mortality from coronary heart disease (CHD) has decreased dramatically in Sweden and other Western countries during recent decades [1]. Researchers in regions including Europe, the United States, and New Zealand have used the previously validated IMPACT mortality model to investigate these trends in many countries in Europe and elsewhere [2–7]. Briefly, the model provides an estimate of the effect of changes in risk factors (e.g., smoking, total blood cholesterol, systolic blood pressure (SBP), physical inactivity, diabetes, body mass index [BMI]) and medical treatments and interventions on past trends in CHD mortality under given scenarios. We can also use the IMPACT model to predict future trends in CHD mortality [8, 9].

Previously, we used the IMPACT model to investigate the decrease in CHD mortality in Sweden between 1986 and 2002 in Sweden [10]. We demonstrated that CHD mortality in the 25–84 year age group decreased by 53.4% in men and by 52.0% in women, and that 13 180 fewer deaths occurred in 2002 than would have been expected if the CHD mortality rates from 1986 had persisted [10]. Based on the IMPACT model, we concluded that approximately one third of the mortality decrease could be explained by increased use of effective medical treatments and interventions, and more than half of the decrease could be explained by population-level improvements in risk factors, particularly a decrease in total blood cholesterol levels (from 6.1 mmol/L to 5.5 mmol/L) [10]. Population studies in the city of Gothenburg show that total blood cholesterol levels in men have decreased from 6.5 mmol/L in 1973 [11] to 5.5 mmol/L in 2002 [10, 12] and in women from 7.4 mmol/L in 1969 [13] to 5.6 mmol/L in 2002 [12]. These decreases in total blood cholesterol likely reflected dietary changes, such as reduced intake of saturated fat and altered fatty acid intake profile. Data for northern Sweden are similar, with a decrease in reported total blood cholesterol from 1986 to 1992 and, after a small increase, a continued decline until 2002, followed by steady levels between 2002 and 2004 [14].

The relationship between diet and total blood cholesterol [15–18] as a major risk factor for CHD is well known [19–21]. Therefore, there have been growing concerns over recent adverse dietary trends in Sweden [14, 22]. However, the possible beneficial health effects of low carbohydrate–high fat diets have been described in very positive terms by the Swedish media [23]. This media debate may have affected food selection among the population, with higher reported intakes of cream, butter, butter-based spreads, and red meat, and lower reported intakes of bread, cereals, and potatoes [14, 22]. Consequently, the reported dietary intake of total fat and saturated fat in the population of northern Sweden has increased markedly since 2002, whereas carbohydrate intake has decreased [14]. In parallel, total blood cholesterol levels have increased since 2003[14], interrupting and even reversing the trend to lower total blood cholesterol levels observed in the 1980s and 1990s. These recent data suggest that a considerable change in dietary fat intake has occurred in the Swedish population since we last applied the IMPACT model to predict CHD mortality based on life style risk factors [10].

In the present study we used the IMPACT model and Swedish lifestyle data to predict how changes in fat intake might affect total blood cholesterol levels and CHD mortality in the Swedish population between 2010 and 2025. We applied two contrasting scenarios for trends in dietary intake of total fat: lower saturated fat intake, which would be a reduction based on current population intake, in a diet based on the Nordic Nutrition Recommendations; and higher saturated fat intake, compared to current population intake, in a low carbohydrate–high fat diet.

## Methods

### The IMPACT model

The IMPACT model was developed to explain changes in trends in CHD mortality in terms of changes in CHD risk factors in the population, and changes in medical management and treatment uptake. The model has been described in detail elsewhere [4, 24]. We used the IMPACT model for the Swedish population aged 25–84 years to evaluate two scenarios with different levels of saturated fat intake, and to compare the results against a baseline scenario where there is no change in risk factors until 2025. The two scenarios were as follows:

1. *a low saturated fat diet scenario* with a decrease in intake of saturated fat equivalent to 10% of total energy (10 E%), which adheres to the current Nordic Nutrition Recommendations [25]; and
2. *a high saturated fat intake scenario* with an increase in intake of saturated fat equivalent to 20 E%, which aims to mimic recent dietary trends in northern Sweden, but corresponds to the 95% percentile values in a recent nationwide survey of the Swedish diet [26].

In both scenarios, we assumed that recent positive trends in other major risk factors would continue. That is, we assumed that by 2025 there would be a 10% (1 g/day) reduction in salt intake, a 5% reduction in smoking, and a 5% decrease in physical inactivity in each 10-year age group. The primary outcome measure was the decline in mortality in 2025 relative to 2010, that is, the total number of deaths prevented or postponed as a result of changes in the risk factors in each scenario. The number of deaths prevented or postponed was defined as the difference between the number of deaths from CHD expected in 2025 and the number of deaths from CHD observed in 2010.

### Baseline scenario: estimating the expected number of deaths from CHD in 2025

For the baseline scenario, we assumed that CHD mortality would continue to decline at the same rate as that recorded for the past two decades. Therefore, we fitted a series of exponential decay models to age-specific CHD mortality rates from 1986 to 2010. The rates were extrapolated to 2025, and multiplied by the official population projections.

### Modelling effects of risk factors

The IMPACT model considers SBP, total blood cholesterol, smoking, and physical inactivity as risk factors for CHD mortality. To model the effect of saturated fat and salt intake on CHD mortality, first we translated these factors into changes in total blood cholesterol and SBP, respectively.

#### Translating changes in saturated fat intake into changes in cholesterol

We calculated the effect of saturated fat intake on total blood cholesterol using the equations reported by Clarke et al [27]. These equations were obtained from metaregression analysis of more than 90 metabolic ward studies, and translate a change in saturated fat intake into a change in total blood cholesterol levels at an isocaloric replacement with polyunsaturated and monounsaturated fats. A decrease in saturated fat intake of 1% corresponds to a decrease in total blood cholesterol of 0.078 mmol/L or 0.048 mmol/L when replaced by polyunsaturated or monounsaturated fat, respectively. In the present model, we assumed that every percent unit of saturated fat intake was replaced by 90% polyunsaturated fat and 10% monounsaturated fat. The analysis indicated that a decrease in saturated fat intake of 1% would result in a reduction in total blood cholesterol of 0.3 mmol/L (i.e., (0.078 × 0.90) + (0.048 × 0.10); (http://www.escardio.org/static_file/Escardio/EU-Affairs/chd-mortality.pdf). We assumed the effect to be linear within the expected range of saturated fat intake (10–20 E%).

#### Translating changes in salt intake into changes in SBP

We modelled the link between salt intake and blood pressure based on data from a Cochrane Database System Review [28]. According to this extensive metaregression, a 6 g/day reduction in salt intake would result in a 7.2 mm Hg reduction in SBP in people with hypertension, and a 3.6 mm Hg reduction in people with normal blood pressure. In 2010 about 37% (weighted average) of the adult Swedish population had hypertension, and we assumed this rate would remain constant for the forecast period. The analysis indicated that a reduction in salt intake of 1 g/day (10%) would result in a reduction in SBP of 0.82 mm Hg (i.e., [(7.2 × 0.37) + (3.6 × 0.63)]/6) across the adult Swedish population.

#### Translating changes in risk factors into changes in CHD mortality

We used two approaches to calculate the number of deaths prevented or postponed from changes in risk factors in the IMPACT model: the regression approach; and the change in the Population Attributable Risk Fraction (PARF). We used the regression approach for total blood cholesterol and SBP. We multiplied the number of CHD deaths in 2010 by the absolute change in risk factor level (defined below), and by an age and gender specific regression coefficient quantifying the estimated relative change in CHD mortality that would result from a change in the risk factor level of one unit (i.e., per 1 mm Hg change in SBP and per 1 mmol/L change in total blood cholesterol (S1 and S2 Tables). We used the PARF for smoking and physical inactivity. We calculated the PARF, which reflects the reduction in mortality rate from CHD if exposure were eliminated, according to the conventional formula: PARF = [P × (RR − 1)]/[1 + P × (RR − 1)] where P is the age and gender-specific prevalence of the risk factor, and RR is the age and gender-specific relative risk for CHD mortality associated with risk factor presence. We then estimated the number of deaths prevented or postponed as the expected CHD deaths in 2025 multiplied by the difference in PARF for 2010 and 2025. Relative risks for smoking and physical inactivity are given in S3 Table and S4 Table, respectively. We rounded all values for the number of deaths prevented or postponed to the nearest 5.

### Sensitivity analyses

We quantified the degree of stochastic uncertainty using Monte Carlo simulation implemented with R software. We repeated random draws from specified distributions for the input variables (S5 Table) to iteratively recalculate the model. We calculated the uncertainty intervals based on 10 000 draws taking the 95% uncertainty intervals as the 2.5th and 97.5th percentiles. We randomly extracted input variables taken from external sources (e.g., beta coefficients and relative risk reductions) from specified distributions. Specific distributions used in our analysis are presented in S5 Table. Furthermore, we investigated the sensitivity in the saturated fat intake projections by comparing the effect of changes in saturated fat intake by -5, -10, +5 and +10 E% from current levels stratified by age group and sex (S6 Table).

### Data collection and sources

#### Population data

We obtained population numbers for each 10-year age group (25–84), stratified by sex, for both the baseline (2010) and prediction (2025) years from the national database (Statistics Sweden). We retrieved mortality data for 2010 from the Swedish National Board of Health and Welfare.

#### Population risk factors

We obtained data on measured total blood cholesterol and SBP from the northern Sweden MONICA study and the Prospective Urban and Rural Epidemiological (PURE) Study, Gothenburg (2007–2009). We obtained data on smoking and physical activity from Living Conditions Surveys (ULF), Statistics Sweden (2010). For the analyses, we calculated total blood cholesterol and SBP by weighting the data from northern Sweden (MONICA; 15%) and southern Sweden (PURE; 85%) to reflect the national population distribution. We expressed the data as population mean values within each 10-year age group and sex. Detailed information on data sources, including data for 1986 and 2002, is summarized in Table 1.

**Table 1:** Data sources for population and risk factor estimations.

Type of data	Source
Population numbers 2010	Statistics Sweden
CHD Mortality in 2010	The Cause of Death Register, The Swedish National Board of Health and Welfare
Population Projection numbers in 2025	Statistics Sweden
Risk Factor Levels in 1986	The AMORIS Study (total blood cholesterol), MONICA GOT and Northern Sweden (SBP, hypertension), ULF, Statistics Sweden (smoking, BMI, diabetes and physical inactivity)
Risk Factor Levels in 2002	MONICA GOT and Northern Sweden, INTERGENE (total blood cholesterol, hypertension), MONICA Northern Sweden and INTERGENE Study, the Prospective Population Study of Women in Goteborg (SBP), ULF, Statistics Sweden (smoking, BMI, diabetes and physical inactivity)
Risk Factor Levels in 2010	MONICA Northern Sweden and the PURE* study, Gothenburg (2007–2009) (total blood cholesterol and SBP), ULF**, Statistics Sweden (smoking, BMI and physical inactivity), VEGA database*** (diabetes)

*Prospective Urban and Rural Epidemiological Study

**Living Conditions Surveys

*** Västra Götalands Vårddatabas.

#### Dietary intake

Dietary intake data from the Västerbotten Intervention Programme (VIP) and the northern Sweden MONICA project are combined in the Northern Sweden Diet Database (NSDD) (http://www.biobank.umu.se/biobank/biobank---for-researchers/northern-sweden-diet-database/). A semiquantitative food frequency questionnaire (FFQ) was used to estimate food intake with a 9-level fixed scale from never to ≥4 times per day, and portion sizes are estimated from photographs of staple foods such as meat, fish, and vegetables. We transformed reported intake frequencies into intake per day, and estimated energy and nutrient intake by weighting reported intake frequencies with portion sizes and nutrient content obtained from the database at the National Food Administration, Sweden (www.slv.se). Nutrient data included the intake of total fat, saturated fat, and monounsaturated and polyunsaturated fat expressed as the E% (S7 Table). We obtained data on salt intake from a national population-based study on dietary intake (Riksmaten [The National Diet] 2010–2011) performed by the Swedish National Food Agency in 1 800 randomly selected adults [26]. The estimated mean intake of salt was 10 g/day across all age groups and for both sexes.

## Results

### Trends in risk factors used in the IMPACT model for the period 1986–2010

Based on the information from the MONICA and PURE studies, physical activity improved, and total blood cholesterol fell in Sweden from 1986 to 2002. However, diabetes prevalence and BMI increased (Table 2). In 2010, total blood cholesterol levels were similar to those in 2002, whereas SBP, diabetes prevalence, and BMI had all increased. Smoking prevalence fell progressively from 1986 to 2010. The percentage of people who were physically inactive was slightly higher in 2010 than in 2002. Risk factors by age and sex for 2010 are shown in Table 3.

**Table 2:** Risk factors in Swedish men and women in 1986, 2002, and 2010.

		Year		
	Risk Factor	1986	2002	2010
**Men**				
	Mean total blood cholesterol (mmol/L)	6.15	5.51	5.48
	Smoking prevalence (%)	31.3	17.6	13.2
	Mean SBP (mm Hg)	135.0	132.9	134.3
	Diabetes prevalence (%)	2.8	4.2	6.7
	Mean BMI (kg/m^2^)	24.8*	26.0	26.3
	Physical inactivity prevalence* (%)	15.9	12.7	17.3**
**Women**				
	Mean total blood cholesterol (mmol/L)	6.19	5.51	5.53
	Smoking prevalence (%)	26.6	19.5	15.2
	Mean SBP (mm Hg)	132.7	129.7	130.8
	Diabetes prevalence (%)	2.5	3.4	5.1
	Mean BMI (kg/m^2^)	23.8*	24.8	24.9
	Physical inactivity prevalence* (%)	15.6	10.4	12.0**

*1988

**Method change in 2008.

**Table 3:** Risk factors in Swedish men and women in 2010, presented by age group.

		Age group					
		25–34	35–44	45–54	55–64	65–74	75–84
**Men**							
	Mean total blood cholesterol (mmol/L)	4.98	5.36	5.48	5.56	5.57	5.48
	Smoking prevalence (%)	11.8	10.5	13.8	18.1	15.8	6.0
	Mean SBP (mm Hg)	125.9	126.4	131.7	139.2	145.4	151.3
	Diabetes prevalence (%)	0.9	1.8	4.3	9.4	15.2	18.0
	Mean BMI (kg/m^2^)	25.5	26.7	26.7	26.6	26.5	25.6
	Physical inactivity prevalence (%)	11.4	20.0	19.7	18.7	14.8	20.9
**Women**							
	Mean total blood cholesterol (mmol/L)	4.66	4.92	5.51	5.51	5.8	5.68
	Smoking prevalence (%)	11.3	14.3	24.2	16.7	13.3	6.3
	Mean SBP (mm Hg)	118.3	119.1	126.5	136.9	144.2	153.3
	Diabetes prevalence (%)	0.8	1.4	2.9	5.9	10.4	14.1
	Mean BMI (kg/m^2^)	23.6	24.5	25.4	25.3	25.8	25.2
	Physical inactivity prevalence (%)	9.0	12.8	10.6	9.9	12.0	23.3

### Population trends in reported dietary fat intake for the period 1986–2013

**Fig 1:**
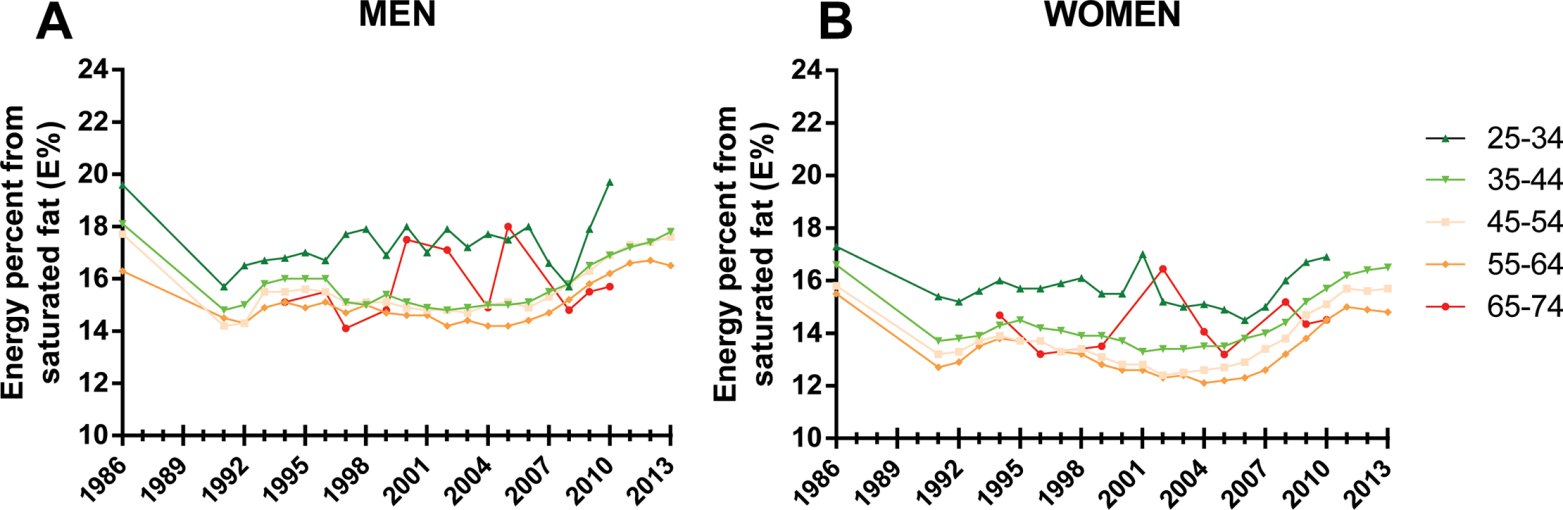
Saturated fat intake in Sweden (1986–2013) by age group and sex. Since 2002–2004 the amount of energy coming from saturated fat as increased in all age groups and in both men (A) and women (B).

Information from the NSDD revealed that the intake of total fat (saturated fat, monounsaturated fat, and polyunsaturated fat) increased by 5 E% from 34.0 E% in 2002–2004 to 39.1 E% in 2011–2013. During the same period, the intake of saturated fat increased from 14.0 E% to 16.7 E%, reflecting increased intake of butter and butter-based spreads, dairy products (cream/crème fraîche), red meat, bacon, and sausages. Butter and butter-based spreads contributed most to the increase in saturated fat intake, increasing from 8.5 E% to 12.9 E%; S7 Table). Fig 1 shows changes in saturated fat intake for men and women in 10-year age groups. In the period from 1995 to 2004, the saturated fat intake (E%) stayed at a fairly constant level of about 15 E% and 13 E%, respectively, in men in women overall, and about 17.5 E% and 15.5 E%, respectively, in young men and women aged 25–34 years (Fig 1). However, between 2005 and 2010, the intake of saturated fat, monounsaturated fat, polyunsaturated fat, and total fat increased in both sexes, but more so in men than in women (S7 Table).

### Prediction of CHD deaths in 2025 based on different lifestyle scenarios

In the baseline scenario (with no estimated change in risk factors), which assumes that recent CHD mortality trends continue, we estimated that about 5 975 CHD deaths might occur in 2025 (4 010 in men and 1 965 in women). This would be 46% lower than the 11 000 deaths expected if the current CHD mortality rates were to remain constant. CHD mortality rates by age and sex for 2010 and 2025 are summarized in S8 Table.

#### Low saturated fat diet scenario with 10 E%

**Fig 2:**
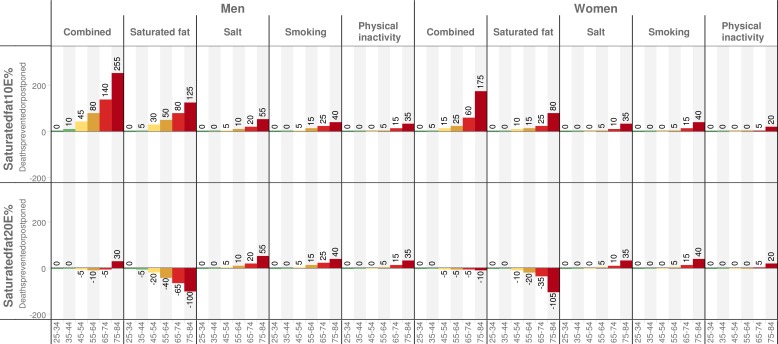
Predicted number of deaths that could be prevented or postponed in men and women from 2010 to 2025 for two diet scenarios. Both scenarios included a reduction in salt intake by 10% and an absolute decrease in physical inactivity and smoking by 5%. Numbers are rounded to nearest 5.

With the low saturated fat diet scenario, a total of 810 (-14%) (minimum estimate 665, maximum estimate 955) deaths could be prevented or postponed in 2025 (535 in men and 275 in women; Fig 2). A reduction of saturated fat intake to 10 E% would result in a reduction in total blood cholesterol of 0.44 mmol/L in men and 0.33 mmol/L in women, and would prevent or postpone approximately 300 deaths in men (minimum estimate 230, maximum estimate 370) and 130 deaths in women (minimum estimate 100, maximum estimate 165). Salt reduction would prevent or postpone 90 deaths in men (minimum estimate 65, maximum estimate 115) and 50 deaths in women (minimum estimate 35, maximum estimate 70). Reduction in smoking might prevent or postpone 90 deaths in men (minimum estimate 40, maximum estimate 140) and 60 deaths in women (minimum estimate 30, maximum estimate 100). Decreases in physical inactivity might prevent or postpone 60 deaths in men (minimum estimate 20, maximum estimate 100) and 30 deaths in women (minimum estimate 10 maximum estimate 50). The number of deaths prevented or postponed according to the four factors, stratified by age and sex, are summarized in Fig 2 and S9 Table.

#### High fat scenario with 20 E% saturated fat

In the high fat scenario, the increased intake of saturated fat would increase total blood cholesterol levels by 0.19 mmol/L in men and by 0.30 mmol/L in women, and result in approximately 400 (+6.7%) additional deaths in 2025. This would represent about 230 additional deaths in men (minimum estimate 175, maximum estimate 285) and 175 additional deaths in women (minimum estimate 130, maximum estimate 220). However, some of these additional deaths would be negated by improvements in other risk factors. Therefore, overall deaths might increase by 20 (minimum estimate −115, maximum estimate 60). There would be about 10 fewer deaths in men (minimum estimate −65, maximum estimate 80) and about 30 additional deaths in women (minimum estimate −85, maximum estimate 15). Reductions in salt, smoking, and physical inactivity might prevent or postpone about 430 (-7.2%) deaths in total (290 deaths in men and 140 in women). Age and sex stratified data for the number of deaths prevented or postponed according to the four factors are summarized in Fig 2 and S9 Table.

#### Sensitivity analysis

In addition to the two scenarios we also compared the effect of absolute changes in saturated fat intake by -10, -5, +5 and +10 E% from current intake as summarized in S6 Table.

## Discussion

Since the 1980s, CHD mortality in Sweden has decreased dramatically, largely reflecting improvements in population risk factors. Changes in diet can powerfully modify total blood cholesterol and blood pressure, two major risk factors for CHD. We developed a model that allowed us to quantify such changes in terms of deaths prevented or postponed. We compared two plausible scenarios: a low diet with just 10 E% from saturated fat (reflecting the Nordic Nutrition Recommendations); and a high fat diet with 20 E% from saturated fat (reflecting recent popular dietary trends, and an increase from the 2010 values of about 17%; Fig 1). We found that adherence to the recommended, lower fat diet would substantially reduce CHD mortality, while the higher fat diet would actually increase the number of CHD deaths.

Trends in total blood cholesterol levels reflect population diet quality. Recent data indicate that the consumption of saturated fat has increased since the mid-2000s, deteriorating to intake levels similar to 1986. If these adverse trends continue, the benefits from continued reductions in smoking and salt intake could be wiped out, with CHD mortality rates levelling off or even increasing in some age groups. The relationship between population total blood cholesterol levels and CHD mortality is well known. However, the powerful roles of “good” and “bad” dietary fats are less well appreciated. In Sweden, there has been a recent increase in the intake of products rich in saturated fat, such as cream, butter, and red meat, with the saturated fat intake rising towards the 20 E% level. This has led to higher total blood cholesterol levels in the population. As seen in Fig 1, consumption of saturated fat in the Västerbotten cohort in 2013 had increased compared with a decade earlier and was close to 20 E% in some age groups (e.g., young men).

In the present study, we particularly focused on one specific risk factor for CHD, dietary saturated fat, and investigated how corresponding changes in blood total blood cholesterol might affect future CHD mortality. We used the previously developed IMPACT model to predict changes in future CHD mortality between 2010 and 2025 in the projected Swedish population aged 25–84. We assumed that the other major risk factors for CHD might continue to improve in line with recent beneficial trends, so that salt intake would fall from 10 g/day to 9 g/day, smoking prevalence would fall from 13% to 8% in men and from 15% to 10% in women, and physical inactivity would decrease from 12% to 7% in men and from 17% to 12% in women.

The Swedish diet has traditionally been high in salt intake [26] with correspondingly high blood pressure levels and more than 25% of the adult population are hypertensive [29]. Reducing salt intake is important from a population perspective, and even a modest reduction of 1 g/day would lead to a considerable decrease in SBP in the population [28]. Furthermore, a 20 mm Hg decrease in SBP is associated with a halving of deaths from stroke and CHD [30, 31]. Even a rather moderate and eminently feasible decrease in salt intake of 1 gram per day would be powerful. Sadly, at present Sweden has no legislation to restrict salt content in food. Conversely, the recent successful salt reduction programme in the United Kingdom decreased consumption by 15%, potentially preventing between 5 000 and 10 000 deaths [32, 33]. We assumed that absolute smoking prevalence would decrease by 5%. Smoking has decreased continuously among the Swedish population since the 1980s. Moreover, a smoking ban in public places was introduced in 2005 and further restrictions in public places are planned. Physical inactivity is an increasing population problem, potentially leading to increased prevalence of overweight, obesity and diabetes. However, awareness of the problem has increased and a 5% decrease in physical inactivity would be achievable through effective, population-wide policies [34–36].

The link between saturated fat intake and CHD mortality remains poorly understood. A recent meta-analysis showed no association between saturated fat and total CHD and a trend for CHD mortality, when results were adjusted for any confounders including LDL cholesterol. However, with a more parsimonious model, that did not include LDL, saturated fat intake was significantly associated with both total CHD and CHD mortality [37]. In addition a large meta-analysis showed a significant effect of reducing saturated fat and/or modifying dietary fat composition on cardiovascular events but not on mortality. The reduction in event was related to the change in total and LDL cholesterol [38].

A recent study from the British Isles used the IMPACT model to project the future CHD mortality assuming that the recent declining trends would continue. One scenario assumed “ideal” improvements in risk factors such as smoking and physical inactivity would decrease by 15% and the intake of dietary salt intake and saturated fat intake up to 30% and to 6% respectively. A second, “modest” scenario assumed absolute reductions in smoking and physical activity by 5% and the intake of dietary salt by 10% intake and a reduction in daily saturated fat intake up to 2 E%. The study showed that achievable reductions in cardiovascular risk factors significantly could reduce the future CHD mortality. Reductions in smoking could decrease CHD deaths by 5.8–7.2%, reductions in physical inactivity by 3.1–3.6% and reductions in salt intake by some 5.2–5.6% while reduction in saturated fat intake would decrease CHD deaths by 7.8–9.0% [9].

However, projections are dependent on the actual risk factor levels. For instance, Sweden has relatively few smokers in the population, with a prevalence of 13.2%. Accordingly we assumed that a 5% absolute reduction in smoking prevalence would be feasible, in contrast to a 15% reduction in the study by Hughes et al [9]. Concerning the saturated fat intake we assumed a daily intake of 10 E% which is according to the Nordic Nutrition Recommendations (low saturated fat intake) [25]. However, current trends show an increasing intake of saturated fat (Fig 1) and accordingly we also projected future CHD mortality based on a high saturated fat intake (20 E%) and changes in risk factors as described above.

The trends in CHD mortality are largely similar in most Western countries, but there are some differences [1]. In particular, trends among younger people show large differences between countries [39–41]. Furthermore, there are differences in smoking, physical inactivity and dietary patterns between countries. In conclusion, models and projections needs to developed and adapted to the specific conditions in the country under study.

### Strengths and limitations

Modelling studies have a number of potential strengths. The best models can integrate and simultaneously analyse huge amounts of data from many sources. However, models are dependent on the extent and quality of the available data. To ensure representativeness all data used in the present study were from Swedish sources such as population studies, national surveys (Statistics Sweden), and governmental reports. In addition, representative data for population risk factors in 1986, 2002, and 2010 were obtained from different parts of Sweden to minimize possible geographical variations, and the data were weighted by population size for sex and for different age groups.

Differences between the periods for diagnostic criteria and the quality of recorded causes of death present a potential limitation to the present study. However, standardized data from population and hospital discharge registries in Sweden are highly accurate and almost 100% complete [42, 39]. The core of the present model was the IMPACT model, which has been validated in a large number of countries [2–7, 10]. The link between changes in lifestyle and changes in risk factors was modelled using data from large meta-analyses of experimental and observational studies. A potential limitation is that the models not directly take into account trends in BMI and diabetes, however by using recent CHD trend data we have indirectly taken into count adverse trends in BMI and diabetes.

We hypothesized that saturated fat would be replaced by polyunsaturated fat (90%) and monounsaturated fat (10%). The change in total blood cholesterol by replacement of saturated fat by polyunsaturated and monounsaturated fat was calculated from a total of 395 analyses on solid food using crossover, parallel, Latin square, and sequential designs [27]. All our assumptions were tested in rigorous sensitivity analyses, which produced reassuringly consistent results.

## Conclusions

An increase in saturated fat intake (up to 20% SFA daily) would increase the total blood cholesterol levels and cancel out the positive effects on CHD mortality from predicted decreases in salt intake, smoking, and physical inactivity. Conversely, small and readily achievable beneficial dietary changes could substantially reduce CHD mortality. Furthermore, substituting “bad” saturated fat (mostly animal), with “good” monounsaturated and polyunsaturated fats (e.g., olive, sunflower, and canola oil) is eminently feasible.

## Supporting Information

S1 Table Systolic blood pressure beta coefficients by age group and sex. (DOCX)

S2 Table Plasma cholesterol beta coefficients by age group and sex. (DOCX)

S3 Table Relative risk in smokers vs non-smokers (95% CI). (DOCX)

S4 Table Relative risk in physical inactive vs sufficient active (95% CI). (DOCX)

S5 Table Specific distributions for model parameters. (DOCX)

S6 Table Deaths prevented or postponed according to changes in saturated fat intake -10, -5,+5 and +10 E%. (DOCX)

S7 Table Daily intake of dietary fat and some selected food groups in men and women. (DOCX)

S8 Table Population numbers and CHD deaths in Sweden in 2010 and predicted population numbers and CHD deaths in 2025 if current trends continue. (DOCX)

S9 Table Deaths prevented or postponed according to the two scenarios. (DOCX)
