# Supplementary material for: Changes in Dietary Fat Intake and Projections for Coronary Heart Disease Mortality in Sweden: A Simulation Study
Source: PLoS One. 2016 Aug 4;11(8):e0160474. doi: 10.1371/journal.pone.0160474 (PMC4973910; doi:10.1371/journal.pone.0160474)
Supplement: S1 Table — (DOCX) [file pone.0160474.s001.docx]

**S1 Table. Systolic blood pressure beta coefficients by age group and sex.**

|  | **Age group (years)** | | | | |
| --- | --- | --- | --- | --- | --- |
| **Systolic blood pressure** | **25-44** | **45-54** | **55-64** | **65-74** | **75+** |
| Men (hazard ratio per 20 mmHg) | 0.49 | 0.49 | 0.52 | 0.58 | 0.65 |
| **Men (log hazard ratio per 1 mmHg)** | **-0.036** | **-0.035** | **-0.032** | **-0.027** | **-0.021** |
| *Minimum* | *-0.029* | *-0.028* | *-0.026* | *-0.022* | *-0.017* |
| *Maximum* | *-0.043* | *-0.042* | *-0.039* | *-0.032* | *-0.025* |
| Women (hazard ratio per 20 mmHg) | 0.40 | 0.40 | 0.49 | 0.52 | 0.59 |
| **Women (log hazard ratio per 1 mmHg)** | **-0.046** | **-0.046** | **-0.035** | **-0.032** | **-0.026** |
| *Minimum* | *-0.037* | *-0.037* | *-0.028* | *-0.026* | *-0.021* |
| *Maximum* | *-0.055* | *-0.055* | *-0.042* | *-0.039* | *-0.031* |
