# Supplementary material for: Changes in Dietary Fat Intake and Projections for Coronary Heart Disease Mortality in Sweden: A Simulation Study
Source: PLoS One. 2016 Aug 4;11(8):e0160474. doi: 10.1371/journal.pone.0160474 (PMC4973910; doi:10.1371/journal.pone.0160474)
Supplement: S2 Table — (DOCX) [file pone.0160474.s002.docx]

**S2 Table. Plasma cholesterol beta coefficients by age group and sex.**

|  | **Age groups (years)** | | | | | |
| --- | --- | --- | --- | --- | --- | --- |
| **Cholesterol** | **25-44** | **45-54** | **55-64** | **65-74** | **75-84** | **85+** |
| Men (mortality reduction per 1 mmol/l) | 0.55 | 0.53 | 0.36 | 0.21 | 0.21 | 0.21 |
| **Men (log mortality reduction per 1 mmol/L)** | **-0.799** | **-0.755** | **-0.446** | **-0.236** | **-0.117** | **-0.083** |
| *Minimum* | *-0.639* | *-0.604* | *-0.357* | *-0.189* | *-0.093* | *-0.067* |
| *Maximum* | *-0.958* | *-0.906* | *-0.536* | *-0.283* | *-0.140* | *-0.100* |
| Women (mortality reduction per 1 mmol/l) | 0.57 | 0.52 | 0.35 | 0.23 | 0.23 | 0.23 |
| **Women (log mortality reduction per 1 mmol/L)** | **-0.844** | **-0.734** | **-0.431** | **-0.261** | **-0.174** | **-0.051** |
| *Minimum* | *-0.675* | *-0.587* | *-0.345* | *-0.209* | *-0.139* | *-0.041* |
| *Maximum* | *-1.013* | *-0.881* | *-0.517* | *-0.314* | *-0.209* | *-0.062* |
