# Supplementary material for: Changes in Dietary Fat Intake and Projections for Coronary Heart Disease Mortality in Sweden: A Simulation Study
Source: PLoS One. 2016 Aug 4;11(8):e0160474. doi: 10.1371/journal.pone.0160474 (PMC4973910; doi:10.1371/journal.pone.0160474)
Supplement: S3 Table — (DOCX) [file pone.0160474.s003.docx]

**S3 Table. Relative risk in smokers vs non-smokers (95% CI).**

|  | **Age groups (years)** | | | | |
| --- | --- | --- | --- | --- | --- |
| **Smoking** | **30-44** | **45-59** | **60-69** | **70-79** | **80+** |
| Men (RR smoker vs non-smoker) | 5.51 (2.47-12.25) | 3.04 (2.66-3.48) | 1.88 (1.70-2.08) | 1.44 (1.27-1.63) | 1.05 (0.78-1.43) |
| Female (RR smoker vs non-smoker) | 2.26 (0.83-6.14) | 3.78 (3.10-4.62) | 2.53 (2.22-2.87) | 1.68 (1.46-1.93) | 1.38 (1.08-1.77) |
