# Supplementary material for: Changes in Dietary Fat Intake and Projections for Coronary Heart Disease Mortality in Sweden: A Simulation Study
Source: PLoS One. 2016 Aug 4;11(8):e0160474. doi: 10.1371/journal.pone.0160474 (PMC4973910; doi:10.1371/journal.pone.0160474)
Supplement: S4 Table — (DOCX) [file pone.0160474.s004.docx]

**S4 Table. Relative risk in physical inactive vs sufficient active (95% CI).**

|  | **Age groups (years)** | | |
| --- | --- | --- | --- |
| **Physical inactivity level** | **15-69** | **70-79** | **80+** |
| Inactive level | 1.71 (1.58-1.85) | 1.50 (1.38-1.61) | 1.30 (1.21-1.41) |
