# Supplementary material for: Changes in Dietary Fat Intake and Projections for Coronary Heart Disease Mortality in Sweden: A Simulation Study
Source: PLoS One. 2016 Aug 4;11(8):e0160474. doi: 10.1371/journal.pone.0160474 (PMC4973910; doi:10.1371/journal.pone.0160474)
Supplement: S5 Table — (DOCX) [file pone.0160474.s005.docx]

**S5 Table. Specific distributions for model parameters.**

| **Group** | **Parameters** | **Distribution** | **Distribution Parameters** |
| --- | --- | --- | --- |
| Population counts in base year and CHD deaths stratified by age and sex | Population counts (no error) | No error (uniform distribution) |  |
|  | CHD mortality (no error) | No error (uniform distribution) |  |
| Population counts in final year stratified by age and sex | Population counts | Normal(mean, SD) | Mean = point estimate; SD = standard error of the mean |
|  | CHD mortality | Normal(mean, SD) | Mean = point estimate; SD = standard error of the mean |
| Prevalence/mean estimates | Prevalence estimates (smoking physical activity, hypertension prevalence) – beta distribution. | Beta (alpha, beta) | Alpha=cases; Beta= non-cases |
|  | Continuous variables (SBP, total cholesterol, salt intake) | Normal(mean, SD) | Mean =point estimate; SD = standard error of the mean |
| Relative risk reduction | Relative risk for CHD deaths for smoking and physical incativity | RelRisk(RR, SE ln(RR)) | RR=relative risk; SE ln(RR) =standard error |
| Beta coefficients | Beata coefficients for quantifying relation of SBP and cholesterol level with CHD mortality | Normal (mean, SD) | Mean = point estimate; SD = standard error of the mean |
