# Supplementary material for: Changes in Dietary Fat Intake and Projections for Coronary Heart Disease Mortality in Sweden: A Simulation Study
Source: PLoS One. 2016 Aug 4;11(8):e0160474. doi: 10.1371/journal.pone.0160474 (PMC4973910; doi:10.1371/journal.pone.0160474)
Supplement: S6 Table — (DOCX) [file pone.0160474.s006.docx]

**S6 Table. Deaths prevented or postponed according to changes in saturated fat intake -10, -5,+5 and +10 E%.**

| **Age and sex** | **Scenario** | **Saturated fat*** | **DPPs [%]** |
| --- | --- | --- | --- |
| **Men & Women 25-84** | -10 E% Saturated fat | 755 (629-886) | 12.6 (11.3-14.0) |
|  | -5 E% Saturated fat | 400 (337-468) | 6.7 (6.0-7.4) |
|  | +5 E% Saturated fat | -444 (-525--370) | -7.4 (-8.3--6.6) |
|  | +10 E% Saturated fat | -930 (-1107--778) | -15.6 (-17.5--13.7) |
| **Men 25-84** | -10 E% Saturated fat | 482 (383-599) | 12.0 (10.3-13.8) |
|  | -5 E% Saturated fat | 256 (202-318) | 6.4 (5.5-7.3) |
|  | +5 E% Saturated fat | -284 (-356--224) | -7.1 (-8.2--6.0) |
|  | +10 E% Saturated fat | -598 (-747--470) | -14.9 (-17.6--12.5) |
| **Women 25-84** | -10 E% Saturated fat | 272 (200-340) | 13.9 (12.0-15.8) |
|  | -5 E% Saturated fat | 144 (107-180) | 7.3 (6.3-8.4) |
|  | +5 E% Saturated fat | -159 (-202--118) | -8.1 (-9.4--6.9) |
|  | +10 E% Saturated fat | -332 (-423--250) | -16.9 (-19.7--14.3) |

DPP, Deaths prevented or postponed. * Data is presented as mean (minimum estimate, maximum estimate)
