# Supplementary material for: Changes in Dietary Fat Intake and Projections for Coronary Heart Disease Mortality in Sweden: A Simulation Study
Source: PLoS One. 2016 Aug 4;11(8):e0160474. doi: 10.1371/journal.pone.0160474 (PMC4973910; doi:10.1371/journal.pone.0160474)
Supplement: S7 Table — (DOCX) [file pone.0160474.s007.docx]

**Table S7. Daily intake of dietary fat and some selected food groups in men and women**

|  |  |  | Year | | | | linear trend | |
| --- | --- | --- | --- | --- | --- | --- | --- | --- |
|  |  |  | 2002-2004 | 2005-2008 | 2009-2010 | 2011-2013 | | p-value |
| **Men** |  | n^a^ | 8,827 | 12,686 | 7,034 | 8,646 | |  |
|  | **Fat intake (E%)** | Total fat | 35.7 (6.5) | 37.0 (6.8) | 39.1 (7.1) | 40.3 (7.3) | | <0.0001 |
|  |  | Saturated fat | 15.0 (3.4) | 15.4 (3.5) | 16.8 (3.9) | 17.5 (4.0) | | <0.0001 |
|  |  | Monosaturated fat | 12.1 (2.6) | 12.6 (2.6) | 13.2 (2.7) | 13.5 (2.7) | | <0.0001 |
|  |  | Polysaturated fat | 5.7 (2.1) | 6.2 (2.4) | 6.2 (2.4) | 6.2 (2.5) | | <0.0001 |
|  | **Daily intake^b^** | Dairy products% | 8.0 (7.1) | 8.5 (7.2) | 8.4 (7.1) | 8.6 (7.5) | | <0.0001 |
|  |  | Butter/butterbased spread | 10.4 (9.8) | 10.7 (10.0) | 13.1 (10.3) | 13.8 (10.1) | | <0.0001 |
|  |  | Cream, crème fraiche | 1.0 (1.3) | 1.2 (1.5) | 1.5 (1.9) | 1.7 (2.0) | | <0.0001 |
|  |  | Red meat | 3.3 (1.8) | 3.4 (2.1) | 3.5 (2.0) | 3.6 (2.1) | | <0.0001 |
|  |  | Bacon/sausage as main meal | 1.5 (1.1) | 1.5 (1.0) | 1.6 (1.1) | 1.6 (1.1) | | <0.0001 |
| **Women** |  | n^a^ | 9,466 | 13,287 | 7,448 | 8,579 | |  |
|  | **Fat intake (E%)** | Total fat | 32.3 (6.0) | 33.8 (6.2) | 36.5 (6.8) | 37.8 (6.8) | | <0.0001 |
|  |  | Saturated fat | 13.1 (3.0) | 13.6 (3.1) | 15.2 (3.6) | 16.0 (3.7) | | <0.0001 |
|  |  | Monosaturated fat | 10.8 (2.2) | 11.3 (2.2) | 12.1 (2.4) | 12.5 (2.4) | | <0.0001 |
|  |  | Polysaturated fat | 5.7 (1.7) | 6.0 (1.9) | 6.1 (1.9) | 6.1 (1.9) | | <0.0001 |
|  | **Daily intake^b^** | Dairy products% | 12.8 (9.7) | 13.3 (9.8) | 13.3 (9.9) | 13.1 (9.8) | | 0.214 |
|  |  | Butter/butterbased spread | 6.9 (8.3) | 8.0 (8.6) | 11.2 (9.7) | 12.3 (9.4) | | <0.0001 |
|  |  | Cream, crème fraiche | 1.2 (1.3) | 1.4 (1.5) | 1.8 (1.9) | 2.2 (2.2) | | <0.0001 |
|  |  | Red meat | 3.2 (1.8) | 3.2 (1.8) | 3.3 (1.9) | 3.4 (1.9) | | <0.0001 |
|  |  | Bacon/sausage as main meal | 1.2 (0.9) | 1.2 (0.9) | 1.3 (1.0) | 1.4 (1.0) | | <0.0001 |

Data are based on FFQ information from the two studies VIP and the Northern Sweden MONICA study.

^a^Mean (SD) values standardized for sex, age, and BMI.

^b^Mean (SD) servings per week standardized for sex, age, and BMI.
