# Supplementary material for: Changes in Dietary Fat Intake and Projections for Coronary Heart Disease Mortality in Sweden: A Simulation Study
Source: PLoS One. 2016 Aug 4;11(8):e0160474. doi: 10.1371/journal.pone.0160474 (PMC4973910; doi:10.1371/journal.pone.0160474)
Supplement: S8 Table — (DOCX) [file pone.0160474.s008.docx]

**Table S8. Population numbers and CHD deaths in Sweden in 2010 and predicted population numbers and CHD deaths in 2025 if current trends continue**

|  |  | **Population** | | **CHD death rates**  **per 100,000** | |
| --- | --- | --- | --- | --- | --- |
|  |  | **2010** | **2025** | **2010** | **2025*** |
| **Men** | 25-34 | 595658 | 673405 | 0.8 | 0.6 |
|  | 35-44 | 653191 | 698391 | 4.7 | 3.2 |
|  | 45-54 | 622753 | 647715 | 35.0 | 17.1 |
|  | 55-64 | 593503 | 644803 | 137.0 | 46.5 |
|  | 65-74 | 460825 | 523658 | 319.0 | 168.3 |
|  | 75-84 | 239134 | 414989 | 1122.0 | 648.9 |
|  | **25-84** | **3165064** | **3602961** | **164.9** | **111.3** |
| **Women** | 25-34 | 566119 | 637131 | 0.4 | 0.2 |
|  | 35-44 | 632114 | 673861 | 1.9 | 1.1 |
|  | 45-54 | 604674 | 636129 | 10.0 | 6.5 |
|  | 55-64 | 593174 | 635401 | 37.0 | 16.6 |
|  | 65-74 | 478846 | 535267 | 123.0 | 63.3 |
|  | 75-84 | 314034 | 465661 | 550.0 | 315.4 |
|  | **25-84** | **3188961** | **3583450** | **81.8** | **54.8** |
| **Total** | **25-84** | **6354025** | **7186411** | **123.2** | **83.1** |

* 2025 CHD death rates and deaths calculated assuming a negative exponential decay model
