# Supplementary material for: Changes in Dietary Fat Intake and Projections for Coronary Heart Disease Mortality in Sweden: A Simulation Study
Source: PLoS One. 2016 Aug 4;11(8):e0160474. doi: 10.1371/journal.pone.0160474 (PMC4973910; doi:10.1371/journal.pone.0160474)
Supplement: S9 Table — (DOCX) [file pone.0160474.s009.docx]

**Table S9. Deaths prevented or postponed according to the two scenarios**

|  |  | **Age** | **All policies together*** | **Saturated fat*** | **Salt*** | **Smoking*** | **Physical inactivity*** |
| --- | --- | --- | --- | --- | --- | --- | --- |
| **Low fat scenario (10 E%)** | **Men** | 25-34 | 2 (1-3) | 2 (1-3) | 0 (0-0) | 0 (0-1) | 0 (0-0) |
|  |  | 35-44 | 10 (0-19) | 7 (0-13) | 0 (0-1) | 2 (0-5) | 0 (0-1) |
|  |  | 45-54 | 44 (16-73) | 32 (11-55) | 3 (1-5) | 7 (2-13) | 2 (0-4) |
|  |  | 55-64 | 79 (33-126) | 52 (21-84) | 8 (3-14) | 14 (4-25) | 6 (1-12) |
|  |  | 65-74 | 142 (75-215) | 79 (41-120) | 22 (11-37) | 24 (6-46) | 17 (2-33) |
|  |  | 75-84 | 257 (174-348) | 126 (87-169) | 55 (36-79) | 40 (0-88) | 37 (0-74) |
|  | **Women** | 25-34 | 1 (0-1) | 0 (0-1) | 0 (0-0) | 0 (0-0) | 0 (0-0) |
|  |  | 35-44 | 3 (2-4) | 2 (1-3) | 0 (0-0) | 0 (0-1) | 0 (0-0) |
|  |  | 45-54 | 13 (6-20) | 9 (4-14) | 1 (1-2) | 2 (1-3) | 1 (0-2) |
|  |  | 55-64 | 24 (9-40) | 13 (5-22) | 3 (1-5) | 6 (2-11) | 2 (0-5) |
|  |  | 65-74 | 58 (27-90) | 27 (13-42) | 10 (4-17) | 15 (4-27) | 7 (1-14) |
|  |  | 75-84 | 176 (115-243) | 81 (54-110) | 36 (21-53) | 39 (9-78) | 20 (1-41) |
| **High fat scenario (20 E%)** | **Men** | 25-34 | 1 (0-1) | 0 (0-0) | 0 (0-0) | 0 (0-1) | 0 (0-0) |
|  |  | 35-44 | -1 (-4-1) | -4 (-8-0) | 1 (0-1) | 2 (0-5) | 0 (0-1) |
|  |  | 45-54 | -7 (-18--1) | -19 (-33--7) | 3 (1-5) | 7 (2-13) | 2 (0-5) |
|  |  | 55-64 | -10 (-28-1) | -38 (-63--16) | 8 (3-14) | 14 (4-25) | 6 (1-12) |
|  |  | 65-74 | -3 (-34-24) | -67 (-102--35) | 22 (11-37) | 24 (6-46) | 17 (3-34) |
|  |  | 75-84 | 31 (-32-96) | -101 (-136--70) | 55 (36-79) | 40 (0-89) | 37 (0-74) |
|  | **Women** | 25-34 | 0 (0-0) | 0 (-1-0) | 0 (0-0) | 0 (0-0) | 0 (0-0) |
|  |  | 35-44 | -2 (-3--1) | -2 (-3--1) | 0 (0-0) | 0 (0-1) | 0 (0-0) |
|  |  | 45-54 | -7 (-13--3) | -11 (-19--5) | 1 (1-2) | 2 (1-3) | 1 (0-2) |
|  |  | 55-64 | -7 (-17--2) | -19 (-32--7) | 3 (1-5) | 6 (2-11) | 2 (0-5) |
|  |  | 65-74 | -5 (-21-8) | -36 (-56--17) | 10 (4-16) | 15 (4-27) | 7 (1-14) |
|  |  | 75-84 | -9 (-57-36) | -104 (-144--70) | 36 (21-54) | 39 (9-77) | 20 (1-40) |

* Data is presented as mean (minimum estimate, maximum estimate)
